# Shotgun sequencing of honey DNA can describe honey bee derived environmental signatures and the honey bee hologenome complexity

**DOI:** 10.1038/s41598-020-66127-1

**Published:** 2020-06-09

**Authors:** Samuele Bovo, Valerio Joe Utzeri, Anisa Ribani, Riccardo Cabbri, Luca Fontanesi

**Affiliations:** 1 Department of Agricultural and Food Sciences, Division of Animal Sciences, University of Bologna, Viale Giuseppe Fanin 46, 40127 Bologna, Italy; 2 Department of Veterinary Medical Sciences, University of Bologna, Via Tolara di Sopra 50, 40064 Ozzano dell’Emilia, Bologna Italy

**Keywords:** Genetics, Genomics, Metagenomics, Biological techniques, Sequencing, Next-generation sequencing

## Abstract

Honey bees are large-scale monitoring tools due to their extensive environmental exploration. In their activities and from the hive ecosystem complex, they get in close contact with many organisms whose traces can be transferred into the honey, which can represent an interesting reservoir of environmental DNA (eDNA) signatures and information useful to analyse the honey bee hologenome complexity. In this study, we tested a deep shotgun sequencing approach of honey DNA coupled with a specifically adapted bioinformatic pipeline. This methodology was applied to a few honey samples pointing out DNA sequences from 191 organisms spanning different kingdoms or phyla (viruses, bacteria, plants, fungi, protozoans, arthropods, mammals). Bacteria included the largest number of species. These multi-kingdom signatures listed common hive and honey bee gut microorganisms, honey bee pathogens, parasites and pests, which resembled a complex interplay that might provide a general picture of the honey bee pathosphere. Based on the *Apis mellifera* filamentous virus genome diversity (the most abundant detected DNA source) we obtained information that could define the origin of the honey at the apiary level. Mining *Apis mellifera* sequences made it possible to identify the honey bee subspecies both at the mitochondrial and nuclear genome levels.

## Introduction

Traditional biomonitoring approaches used to obtain information on living organisms for ecological and epidemiological studies are based on appropriately designed sampling strategies that rely on the direct identification and collection of biological specimens from the organisms under investigation in a defined area or context^1^. These approaches have several limits, including the difficulties of reaching remote locations and impervious places and the prohibitive sampling costs, particularly when many data points and complete inventories of the organisms are needed. The analysis of environmental DNA (eDNA)^2^ coupled with high-throughput sequencing (HTS) technologies has revolutionized this field of investigation, overcoming these problems and limitations^3,4^ and increasing precision and detection sensitivity, compared to traditional approaches^5,6^.

Honey bees are unique large-scale monitoring tools due to their extensive foraging activities and environmental exploration^7,8^. Honey bees get into direct contact with the environment in which they live and bring into their hives, and in turns into the honey, environmental contaminants and traces^7,9^. Therefore, honey bee activities define several features of the honey they produce, making this hive product an interesting collector of eDNA signatures^10^. Honey contains eDNA traces that derive from the pollen, from insects (including the honey bees that produced it), viruses, fungi and bacteria that characterize the hive microbial environment and the honey bee hologenome^11^. This information can be used for honey authentication, determining its entomological, botanical and geographical origin^12–21^. Moreover, this DNA can be used to obtain information related to the honey bee pathosphere^22^. Thus far, honey eDNA has been studied using mainly PCR based metabarcoding approaches coupled to HTS^12,14–17^. These approaches have as main disadvantage of studying only a targeted fraction of DNA, that might also be biased by uneven DNA amplification across organisms^23,24^. Although multiple primer sets targeting different genome regions could be used to overcome these problems^14,25^, shotgun sequencing of all DNA, without any preliminary selection, has the potential to better describe the taxonomic complexity^26,27^, being an untargeted and a PCR-free approach.

Shotgun metagenomics or all DNA shotgun sequencing have been applied to describe viromes and bacteriomes communities for several different applications^28,29^ or eukaryotic unicellular and multicellular complex communities^30–41^. These studies faced the challenging interpretation of the sequencing data, considering that for many organisms the complete genome is not available. In addition, a full characterization of eDNA is possible only if sequencing efforts (i.e. sequencing depth) saturate the whole spectrum of contributing organisms. Unfortunately, this issue represents an unsolved question as sample origin is not always available and metagenome complexity is not trivial to estimate. Moreover, high computational efforts would be needed to process millions of sequencing reads against heterogeneous references. We recently tested the use of a sparse shotgun metagenomic sequencing approach to describe the multi-kingdom signature present in the honey DNA^10^. Computational effort was proportional to the limited number of sequenced reads (hundreds of thousands), that however were enough to detect interesting signatures from arthropods, plants, fungi, bacteria and viruses characterizing the analysed honey samples^10^.

In this methodological study, we applied for the first time a high depth shotgun sequencing approach of honey DNA, combined with a specifically designed bioinformatic pipeline, to describe in more detail the complex ecosystems of the honey bee colony superorganism using its agroecological recovered DNA. Sequence data were mined at different levels, taking advantage from the high sequencing depth that made it possible to recover and reconstruct information that would not be possible otherwise. Compared to our sparse sequencing approach^10^, deep sequencing highlighted three major advantages. First of all, the high depth allowed to strengthen the estimation of taxa abundances. Second, it was possible to capture the within organism genetic diversity that opened the possibility to differentiate honey produced from different apiaries, as in the case of the *Apis mellifera* Filamentous Virus. Last but not least, by capturing and analysing sequence information derived from the honey bee mitochondrial and nuclear genomes it made it possible to identify the *Apis mellifera* subspecies that produced the honey. The obtained results provided new insights on the honey bee hologenome complexity that might be useful for many different applications.

## Methods

### Honey samples, DNA extraction and sequencing

Three polyfloral honey samples (hereafter referred to as HB9, HB12 and HB13) were collected in 2018 directly from three honeycombs, each from a different apparently healthy colony. HB9 was from an apiary in the province of Bologna (Emilia-Romagna, Italy). HB12 and HB13 were from another apiary in the province of Modena (Emilia-Romagna, Italy). Honeycombs were immediately frozen at −80 °C till DNA extraction. For DNA isolation, honeycombs were slowly defrosted at room temperature. Then, honey was separated from the honeycomb using a gravimetric method at room temperature that included a filtering step to eliminate residual materials. Honey was then immediately used for DNA extraction, following the protocol previously described^15–17^. Briefly, honey samples have been pre-treated adding ultrapure water in 50 g of starting material divided in four aliquots of 12.5 g. After vortexing and incubating at 40 °C for 1 minute in order to melt the sugars, the tubes were centrifuged at 5000 g at room temperature for 25 minutes. The resulting supernatant was then discarded and 5 mL of ultrapure water were added in each tube and then the content of the four tubes was merged in a single 50 mL tube. A second centrifugation at 5000 g for 25 minutes at room temperature followed and the supernatant was discarded. The resulting pellet was resuspended in 0.5 mL of ultrapure water and transferred in a 1.5 mL tube containing about 12 glass beads (500 µm) and vortexed for 3 min. The sample was the transferred in a new 1.5 mL tube removing the beads and stored at 4 °C. DNA extraction was performed using 1 mL of CTAB buffer [2% (w/v) cetyltrimethylammoniumbromide; 1.4 M NaCl; 100mMTris-HCl; 20 mM EDTA; pH 8], with the addition of 5 µL of RNase A solution (10 mg/mL) and 30 µL of proteinase K solution (20 mg/mL). Tubes were then incubated at 65 °C for 90 minutes mixing gently, and centrifuged for 10 minutes at 16000 g. A total of 700 µL of the obtained supernatant was transferred in a new tube containing 500 µL of chloroform/isoamyl alcohol (24:1) solution, vortexed for 30 seconds and then centrifuged at 16000 g for 15 minutes at room temperature. The supernatant was transferred in a new 1.5 mL tube and the DNA was isolated and purified in two steps, with isopropanol and then ethanol 70%. DNA was finally resuspended with 30 µL of sterile H_2_O and stored at −20 °C.

Extracted DNA was quality checked with a TBE 1% agarose gel and the concentration was measured using Qubit 2.0 fluorimeter (Thermo Fisher Scientific, Waltham, MA USA). This quality control analysis evidenced that the extracted DNA from all honey samples was degraded, as expected, confirming previous evaluations^10,15^.

Three genomic libraries were constructed and sequenced on a BGISeq500 machine, following the provider’s protocol, obtaining paired-end reads of 100 bp in length with an inner distance of 170 bp. Data were quality checked using FASTQC v.0.11.7 (https://www.bioinformatics.babraham.ac.uk/projects/fastqc/). No other filtering procedures were adopted.

### Metagenome assembly and taxonomic assignment of sequenced reads

Reads were assembled with MEGAHIT v.1.1.3^42^ with default parameters except the “*meta-large*” option that forced the usage of a *k*-mer list equal to 27,37,47,57,67,77,87. A three-metagenome co-assembly was generated.

Three different annotation resources were used for taxonomic assignment: (i) the National Center for Biotechnology Information nucleotide database (NCBI nt^43^, downloaded from ftp://ftp.ncbi.nlm.nih.gov/blast/db/FASTA/nt.gz; ~50 million entries; August, 2019), (ii) the HoloBee database v2016.1, section HoloBee-MOP (69 entries), comprising mostly of chromosomal, mitochondrial and plasmid genome assemblies and aggregating as much honey bee holobiont genomic sequence information as possible (HB_Mop_v2016.1^44^; https://data.nal.usda.gov/dataset/holobee-database-v20161) and (iii) a custom database that comprised the *A. mellifera* Amel_HAv3.1 reference genome (GCF_003254395.2) and the latest version of the genome of several honey bee enemies, parasites and pathogens as retrieved from the NCBI Genome database [*Aethina tumida* (GCF_001937115.1)*, Galleria mellonella* (GCF_003640425.1)*, Varroa desctructor* (GCF_002443255.1)*, Ascosphaera apis* (GCA_001636715.1)*, Nosema apis* (GCA_000447185.1)*, N. ceranae* (GCF_000988165.1)*, Crithidia mellificae* (GCA_002216565.1)*, Lotmaria passim* (GCA_000635995.1)*, Melissococcus plutonius* (GCF_004001225.1)*, Paenibacillus larvae* (GCF_002951935.1) and *Apis mellifera* Filamentous Virus (GCF_001308775.1)]. BLAST + v.2.7.1^45^ (algorithm *blastn*, default parameters) was used to align assembled DNA sequences (commonly called contigs) on the sequence databases. For each assembled contig, we retained all the alignments presenting an E-value ≤ 0.01, sequence coverage ≥ 50% and a sequence identity ≥ 75%. These settings followed our previous work^10^ as a grid search here performed did not point out any optimal threshold in sequence coverage and identity. Moreover, coverage was lowered to 50% to account for assembly errors and database incompleteness. For contigs presenting more than one alignment over different entries, we considered the whole set of hits presenting statistics (i.e. sequence coverage, sequence identity and E-value) equal to the top one (lowest E-value). Taxonomic assignment followed then the Lowest Common Ancestor (LCA) approach considering the organismal division provided by GenBank (i.e. Bacteria, Primates, Rodents, Other mammals, Other vertebrates, Invertebrates, Plant and Fungi, Viruses, Phages, Structural RNA sequences, Synthetic and chimeric sequences, Unannotated sequences)^46^. The LCA procedure was implemented in Python 2.7 by using the graph library NetworkX v.2.0 (https://networkx.github.io/). Lastly, we considered as reasonably annotated contigs presenting the above reported statistics (E-value ≤ 0.01, coverage ≥ 50% and identity ≥ 75%) while interrogating the databases in the following order: NCBI nt, HB_Mop_v2016.1 and our custom database.

**Figure 1:**
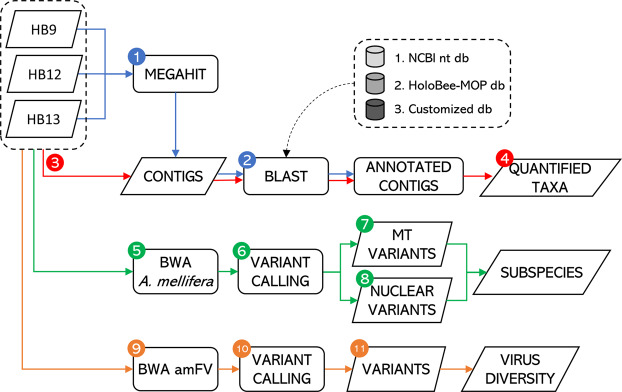
Flowchart of the bioinformatic pipeline used to characterize the hologenome and environmental signature of the honey bee colony superorganism detected from honey DNA. Steps are as follows: (1) assembly of sequenced reads via MEGAHIT; (2) taxonomical assignment via BLASTN; (3) remapping of sequenced reads over the assembled contigs in order to (4) quantify the detected organisms; (5) mapping of sequenced reads via BWA over the *Apis mellifera* reference genome; (6) detection of genome variants; (7) subspecies identification through inspection of the mitochondrial DNA (mtDNA; method described by Utzeri *et al*.^15^; (8) subspecies identification via inspection of nuclear single nucleotide polymorphisms (SNPs; panel proposed by Muñoz *et al*.^60^); (9) mapping of sequenced reads via BWA over the *Apis mellifera* filamentous virus; (10) detection of genome variants; (11) estimation of virus diversity (Fixation index computation).

Reads were mapped back on the assembled contigs with BWA-MEM tool v.0.7.17^47^ (default parameters) to quantify the abundance of each detected taxon. The number of mapped reads and the depth of sequencing of each contig was computed for each sample, separately. Figure 1 describes the flowchart of the bioinformatic pipeline used to characterize sequencing data obtained from the analysed honey samples, with information from the subsequent specific bioinformatic descriptions and statistical analyses.

### Assessing genetic diversity of *Apis mellifera* Filamentous virus populations

As *Apis mellifera* Filamentous Virus (AmFV) had the genome with the highest depth of sequencing in all three honey samples, sequence data for this virus were further analysed. Reads were re-mapped on the AmFV genome with BWA, discarding reads presenting a mapping quality lower than 20. SAMtools v.1.7^48^ (default parameters) was used to call biallelic single nucleotide polymorphisms (SNPs). Only SNPs having at least three reads for the alternative allele were retained for further analyses. This approach is commonly accepted to discard sequencing errors and as a quality control approach^48^. For each honey sample and for each polymorphic site, alternative nucleotide (allele) frequencies were estimated by counting the number of reads having the two alternative forms. Genetic distance between pairs of AmFV populations (i.e. obtained from two different honey samples in pairwise analysis) was estimated by computing Fixation index (F_ST_) values for each detected SNP, as described by Karlsson *et al*.^49^. For each comparison, the F_ST_ values were computed and averaged over the interrogated SNPs. Pipelines were developed in Python 2.7.

### Comparative taxonomic analyses of sequencing data

Sequencing efforts were evaluated by means of rarefaction curves. Briefly, for each honey sample the average number of taxa was plotted as a function of the percentage of remapped reads, randomly sampled without replacement. Ten different sets of randomly sampled reads were used to compute the average number of the detected taxa.

To take in to account the compositional nature of the data (reads counts have an arbitrary total imposed by the instrument), the centered log-ratio (clr) transformation was applied to each profile as $$clr({\boldsymbol{x}})={\boldsymbol{y}}=({y}_{i},\,\ldots ,{y}_{D})$$$$=({\log }_{10}\frac{{x}_{i}}{g({\boldsymbol{x}})},\ldots \,,\,{\log }_{10}\frac{{x}_{D}}{g({\boldsymbol{x}})})$$, where ***x*** is a vector of read counts of size D and $$g(x)=\sqrt[D]{\mathop{\prod }\limits_{i}^{D}{x}_{j}}$$ is its geometric mean^50–53^. Then, similarity of honey taxonomic profiles was assessed by two different measures. The first one was the Aitchison’s distance^53^, equivalent to the Euclidean distance between centred log-ratio transformed compositions. Aitchison’s distance was computed as $$d({{\boldsymbol{y}}}_{{\boldsymbol{i}}},{{\boldsymbol{y}}}_{{\boldsymbol{j}}})=\sqrt{\mathop{\sum }\limits_{i}^{D}{({y}_{ii}-{y}_{ji})}^{2}}$$, where ***y***_***i***_ and ***y***_***j***_ are the centred log-ratio transformations of the i^th^ and j^th^ taxonomical profile. The second measure was the proportionality index *ρ*^50^, computed as $$\rho ({{\boldsymbol{y}}}_{{\boldsymbol{i}}},{{\boldsymbol{y}}}_{{\boldsymbol{j}}})=1-\frac{var({{\boldsymbol{y}}}_{{\boldsymbol{i}}}-{{\boldsymbol{y}}}_{{\boldsymbol{j}}})}{var({{\boldsymbol{y}}}_{{\boldsymbol{i}}})+var({{\boldsymbol{y}}}_{{\boldsymbol{j}}})}$$.

Equally abundant organisms were detected by computing the signed version of the coefficient of variation as $CV{ \% }_{i}=\frac{{\sigma }_{i}}{|{\mu }_{i}|}\times 100$, where, for the i^th^ organism, σ_i_ and |μ_i_| represent the standard deviation and the absolute value of the mean. Organisms having a CV% < 10 were considered equally abundant.

Similarity analysis and coefficients of variation were computed in R 3.6.0^54^.

### Mining metagenomic data to extract information on the subspecies of *Apis mellifera*

To further test the usefulness of the sequencing data, obtained reads were evaluated to extract information that could be used to assign organisms at the subspecies level. Within the *A. mellifera* species, lineages have been first defined at the morphological level; followed DNA based classification schemas that relied on mitochondrial DNA and more recently on nuclear DNA. These approaches were then combined to define sub-species features^55–61^. The *A. mellifera* mitochondrial lineage was initially detected *in silico* by analysing the mitochondrial DNA (mtDNA) informative region used in the PCR based assay developed by Utzeri *et al*.^15^. Briefly, the mtDNA haplotype variability of the mitochondrial genome region NC_0015661:3363–3447 was inspected to discriminate the A, C and M honey bee lineages. The *in silico* read-based analysis was followed by the PCR analysis carried out using an aliquot of the same DNA extracted from the three honey samples used as template. This *in vitro* analysis was carried out by applying the protocol described by Utzeri *et al*.^15^.

Then, we attempted the mining of *A. mellifera* subspecific nuclear DNA information extracted from the metagenome. In this approach, we made use of: (i) a set of 144 ancestry-informative marker SNPs proposed by Muñoz *et al*.^60^ and (ii) allele frequencies (AF) for 117 of these SNPs provided for different *A. mellifera* subspecies and populations (*A. m. carnica*, *A. m. ligustica*, *A. m. mellifera*, Buckfast, different hybrids and artificial DNA pools of DNA from different subspecies) by the works of Muñoz *et al*.^60^ and Henriques *et al*.^62^. BLAST+ was used to locate SNPs on the latest version of the *A. mellifera* reference genome (Amel_HAv3.1; downloaded from the NCBI resource) by mapping the related DNA probes. We considered properly mapped nucleotide probes presenting a sequence identity >97% and an E-value <0.01. DNA probes mapping on two or more genome positions were discarded (no. = 0). Transversions (GC ↔ CG and AT ↔ TA) were further discarded (no. = 11)^63^. We obtained a dataset of 106 SNPs and AFs available for the 22 honey bee populations/pools reported in Muñoz *et al*.^60^ and Henriques *et al*.^62^ (Supplementary Table S1). SNPs were further evaluated mining the sequenced reads as follows: (i) reads were mapped with BWA-MEM (default parameters) on the Amel_HAv3.1 genome, (ii) duplicated reads were removed with Picard v.2.1.1 (https://broadinstitute.github.io/picard/) and (iii) properly paired reads presenting a mapping quality Q > 20 were used for SNP calling with SAMtools. In each sample, AFs were estimated counting the number of reads presenting the reference and the alternative allele.

Identification of the *A. mellifera* subspecies was carried out measuring the genetic distance between the analysed samples and the different populations/pools used as references and retrieved from the study of Muñoz *et al*.^60^. Briefly, vectors of AFs were used to compute a dissimilarity matrix ***D*** in which each value represents the Euclidean distance *d* between two populations, computed as $d({\boldsymbol{p}},{\boldsymbol{q}})=\sqrt{\mathop{\sum }\limits_{i=1}^{n}{({q}_{i}-{p}_{i})}^{2}}$, where ***p*** and ***q*** are vectors of AFs of dimension *n* = 106. Allele frequencies were studied with a heatmap, in which the ***D*** matrix was exploited to perform hierarchical clustering (Ward’s distance was used) and multidimensional scaling (MDS). The subspecies attributed to the *A. mellifera* DNA retrieved from the honey metagenome was the one presenting the lowest Euclidean distance. In a second approach, in order to deal with uncertainty in frequency estimation, we shrank to AF = 0.5 those AFs presenting a value different from zero or one. Reliability of these two approaches was assessed by measuring the trend to endow random samples (no. = 10,000) with a specific *A. mellifera* subspecies. In each random sample, the AF of the i^th^ SNP was sampled (with replacement) from the related AF distribution based on the 22 populations/pools of Muñoz *et al*.^60^ and Henriques *et al*.^62^.

Analyses were carried out in R by using the function *dist*, *heatmap.2* and *cmdscale*, for distance, heatmap/clustering and MDS computation, respectively.

## Results

### Sequencing data, metagenome assembly and taxonomic assignments

The number of reads that has been obtained and then considered for this shotgun DNA sequencing analysis of the investigated honey samples is reported in Table 1. We produced a total of 90,941,469 of 100-bp length read pairs, with an average number per sample of ~30.3 million of read pairs.

**Table 1:** Sequenced, aligned and annotated reads from the analysed honey samples.

ID^a^	No. of reads		
	Sequenced	Mapped (%)^b^	Annotated (%)^c^
HB9	60,882,286	57,852,111 (95%)	40,547,256 (67%)
HB12	60,247,576	56,323,607 (93%)	29,737,907 (49%)
HB13	60,753,076	583,72,652 (96%)	36,318,137 (60%)

^a^Honey sample internal identification number.

^b^No. of reads mapped back on the assembled contigs. Percentage is given in relation to sequenced reads.

^c^No. of reads mapped back on the annotated assembled contigs. Percentage is given in relation to sequenced reads.

**Figure 2:**
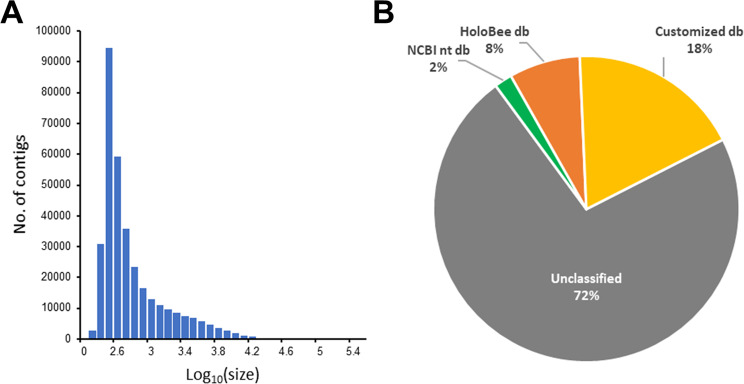
(**A**) Contig length distribution (data are presented in Log_10_ scale). (**B**) Contigs annotated with the different reference databases.

Reads were assembled with MEGAHIT into 341,370 contigs, for a total 417,127,619 assembled DNA bases. Contig size ranged from 200 bp to 251,782 bp, with an average size of 1,222 bp. The N50 parameter (length of the median contig, representing the length of the smallest contig at which half of the assembly is represented^64^) was equal to 3,156 bp. Figure 2A reports the distribution of assembled contig length.

We taxonomically annotated a total of 94,179 contigs (28%) as following: (i) 6,451 considering the NCBI nt resource, (ii) 25,630 based on the HoloBee-MOP resource and (iii) the remaining 62,098 through the customized database containing the sequence of several relevant genomes, as described in Materials and methods. Figure 2B shows the percentage of annotated contigs using the different database resources. The LCA algorithm was applied to solve taxa assignment of 1,537 contigs (1.6%). Annotated sequences ranged from 200 up to 180,460 nucleotides.

### Re-mapping reads on assembled contigs

A total of 172,548,370 single reads, representing ~95% of the whole set of sequenced reads, was mapped back to the assembled contigs (Table 1). This percentage decreased to 49–67% for the three samples (no. of reads = 106,603,300) when considering only annotated contigs (Table 1). Rarefaction curves reached the plateau for all the three analysed samples (Supplementary Fig. S1).

The average read depth of the 341,370 assembled contigs was in the range 0.08× – 144,366× (median = 4×). Reads were not mapped back for 8 contigs. Considering only the 94,179 annotated contigs (Supplementary Table S2), read depth ranged from 2× to 1,865×, for *Crithidia mellificae* (a trypanosomatid parasite of *A. mellifera*) and *Apis mellifera* Filamentous Virus (an almost ubiquitous virus with mild or not yet completely defined pathogenetic effects on honey bees^65,66^), respectively.

### Identification of the most represented organisms

Contigs were assigned to a total of 191 organisms spanning different classes of the GenBank organismal division^46^ (Table 2; Supplementary Table S2). About 55% of them presented at least one contig with sequence identity (SI) > 90%, ~42% had at least one contig with SI ≥ 95% whereas ~20% was annotated only with contigs having SI < 80% (Supplementary Table S2).

**Table 2:** Number of reads obtained for the analysed honey samples belonging to the different taxonomical levels (defined following the organismal division provided by GenBank).

ID^a^	No. of reads							
	Bacteria (no. = 142)^b^	Plants and Fungi (no. = 26)	Invertebrates^c^ (no. = 17)	Viruses (no. = 1)	Phages (no. = 2)	Mammals^d^ (no. = 1)	Rodents (no. = 1)	Other^5^ (no. = 1)
HB9	16,410,099	23,251	22,579,998	1,533,252	166	18	262	210
HB12	19,049,125	62,172	1,684,216	8,939,626	582	111	2,004	71
HB13	14,830,345	22,008	543,139	20,911,867	9,996	356	261	165
Total	50,289,569	107,431	24,807,353	31,384,745	10,744	485	2,527	446

^a^Honey sample internal identification number.

^b^No. of detected organisms.

^c^GenBank group that also includes arthropods and protozoan species^46^.

^d^Non rodent mammals^46^.

^e^Environmental samples^46^.

**Figure 3:**
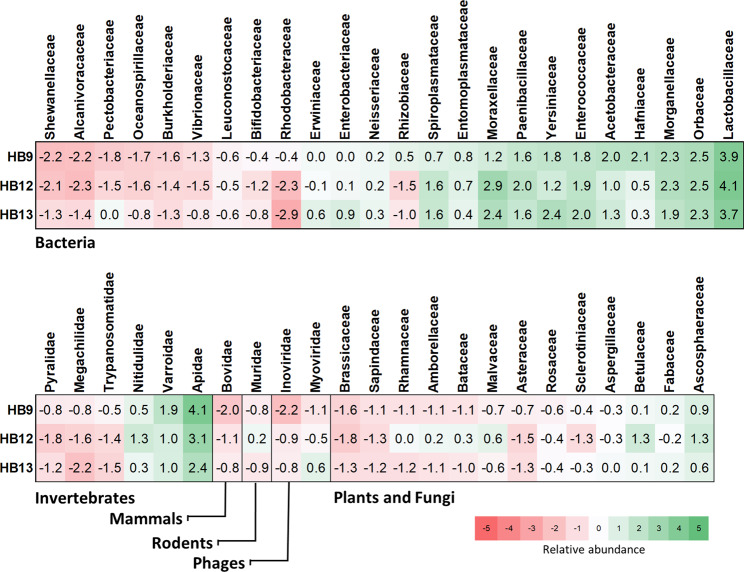
Heatmap of the relative abundance (centered log-ratio transformed read counts) of the different families recovered from the metagenome sequencing of the three analysed honeys. Data are stratified by organismal class and ordered using the reference values of honey sample HB9.

Considering the number of different organisms representing each organismal division^46^, the most represented taxonomical class was Bacteria (no. = 142; 74%) followed by Plants and Fungi (no. = 26; 14%) and Invertebrates (no. = 17; 9%). The remaining 6 organisms (3%) encompassed other taxonomical groups (Environmental samples, Phages, Rodents, non-Rodent Mammals and Viruses). Based on the number of remapped reads, Bacteria represented 47% of all annotated reads, followed by Viruses (29%) and Invertebrates (23%, see also below). For about 72% of cases, organisms were assigned at least at the level of species (~37% of them presented at least one contig with SI ≥ 95%) whereas for 19% of the taxonomical assigned elements, a lower taxonomical rank was not indicated (Supplementary Table S2). Relative abundance of the different groups at the family levels(is presented in Fig. 3.

The taxonomic similarity among samples was assessed using the centered log-ratio transformed read counts. The Aitchison’s distance was equal to 14.3, 14.8 and 9.2 for HB9 *vs* HB12, HB9 *vs* HB13 and HB12 *vs* HB13 comparisons respectively, denoting a marked similarity between the samples HB12 and HB13. This similarity was also confirmed by means of the proportionality coefficient *ρ*, which ranges from −1 (perfect reciprocality) to +1 (perfect proportionality)^50^. Values were equal to 0.71, 0.63 and 0.87 for HB9 *vs* HB12, HB9 *vs* HB13 and HB12 *vs* HB13 comparisons, respectively. The relationship of read counts between honey samples is shown in Supplementary Fig. S2.

### Invertebrates

The group Invertebrates defined in the GenBank classification includes two main taxonomic groups (Arthropoda and Euglenozoa). Within the Invertebrates group (Supplementary Table S2), the genus *Apis* had the largest number of mapped reads. This genus accounted for about 23% of the total sequenced reads and 98% of all reads assigned to arthropods. *A. mellifera* contigs, with an average sequence identity (SI_M_) to the Amel_HAv3.1 reference genome greater than 98.5%, accounted for 99.5% of all reads assigned to the genus *Apis*. The remaining 0.5% included reads matching *A. cerana*, *A. dorsata*, *A. florea*, *A. cerana cerana* and *A. m. carnica* sequences. Other species of the *Apoidea* superfamily were captured by this shotgun analysis, such as *Habropoda laboriosa*, *Bombus impatiens*, *B. terrestris* and *Megachile rotundata*. However, those organisms were probably identified due the high homology of their genome with parts of the *A. mellifera* genome (SI_M_ < 98%).

Sequencing reads identified also other arthropods and a few trypanosomatids (Euglenozoa) presenting a SI_M_ value in the range of 85–95% and including honey bee parasites and pests such as *Varroa destructor* (the most important honey bee parasite), *Aethina tumida* (the small hive beetle, which is a free-living predator and scavenger affecting bee populations) and *Galleria mellonella* (the greater wax moth or honeycomb moth), among the Arthropoda, and *Lotmaria passim* and *Crithidia mellificae*, among the Euglenozoa.

### Plants and Fungi

*Betula pendula* was the plant species presenting the largest number of mapped reads (4.5% of the total realigned reads; SI_M_ > 97.5%). Several other plant species belonging to different plant families were identified with a certain degree of sequence identity (Supplementary Table S2). Among the most represented families, we had *Fabaceae* with several species of the genus *Medicago* (SI_M_ in the range 90–98%), Malvaceae with the *Bombax ceibe* species (SI_M_ > 92%), Rhamnaceae with the *Ziziphus jujube* (SI_M_ > 92%) and Rosaceae represented by several species of the genus *Prunus* (SI_M_ in the range 95.5–99.6%).

Six different species of fungi (*Aspergillus flavus*, *Sclerotinia sclerotiorum*, *Rhynchosporium orthosporum*, *Aspergillus japonicus*; *Penicillium sp. ShG4C* and *Ascosphaera apis;* Supplementary Table S2), classified as pathogens for either honey bees or for plants or humans, were detected. *Penicillium* species might play also a beneficial role due to their potential antimicrobial productions. Their degree of SI_M_ ranged from 84% to 98%. *Ascosphaera apis*, the agent of the chalkbrood disease, accounted for the largest number of assembled contigs (no. = 48) and mapped reads (about 40,000) among all fungi. *Aspergillus flavus*, the agent of the stonebrood disease, was the second most represented fungus species (Supplementary Table S2).

### Bacteria

A total of 142 taxa were identified within the Bacteria (Supplementary Table S2), representing 24 different bacterial families. For sake of clarity, we will now present the most represented bacterial organisms by dividing them into three different categories: honey bee non-pathogenic cobiont bacteria, honey bee pathogenic cobiont bacteria and other bacteria that are part of the honey bee hologenome complex^11^.

#### Honey bee non-pathogenic cobiont bacteria

*Lactobacillus* represented the most abundant genus among the detected bacteria (88% of the bacterial reads), with the largest part of reads assigned to the environmental non-pathogenic cobiont species *Lactobacillus kunkeei)*. Followed the genus *Gilliamella* (2% of the bacterial reads), with the largest part of reads assigned to *G. apicola*, another gut symbiont of honey bees. Several other non-pathogenic bacterial cobionts included *Frischella perrara, Snodgrassella alvi*, *Bifidobacterium asteroids, Parasaccharibacter apium*, *Leuconostoc mesenteroides* and *Rahella aquatilis*.

#### Honey bee pathogenic cobiont bacteria

Several other bacteria, recognized as honey bee pathogens, were detected in all honey samples. These included *Melissococcus plutonius* (the aetiological agent of the European foulbrood disease)*, Paenibacillus larvae* (determining the American foulbrood disease), *Hafnia alvei* (one of the most frequently recovered members of the family Enterobacteriaceae in the gastrointestinal tract, component of the normal fecal honey bee microbiota and in some cases shown to have pathogenetic roles in infections), *Spiroplasma melliferum* and *S. apis* (two pathogenic spiroplasmas detected in honey bee colonies showing different disease symptoms, including “May disease” for *S. apis*^67^).

#### Other bacteria

The genus *Acinetobacter* accounted 29 different species (15.0% of the bacterial reads), even if their identification seems not so reliable since the SI_M_ was in the range 75.0–87.0%. The genus *Spiroplama* (SI_M_ in the range 76.0–99.8%) accounted for the 1.4% of the bacterial reads. Within this genus we detected the above mentioned honey bee pathogens *S. melliferum* and *S. apis* and several plant pathogens including *S. citri*, *S. phoeniceum* and *S. kunkelii*, and *S. diminutum*. *Arsenophonus nasoniae*, the son-killer bacterium of the parasitic wasp *Nasonia vitripennis*, was the third most represented bacteria detected in this study (SI_M_.= 93%). As fourth most abundant bacteria (4.8% of the bacterial reads), we detected *Serratia symbiotica (*SI_M_ = *98.81%)*, a symbiont of aphids. Other bacteria including potential human pathogens were detected with SI_M_ > 96%, such as the genus *Cedecea* and *Klebsiella*.

### Viruses

**Figure 4:**
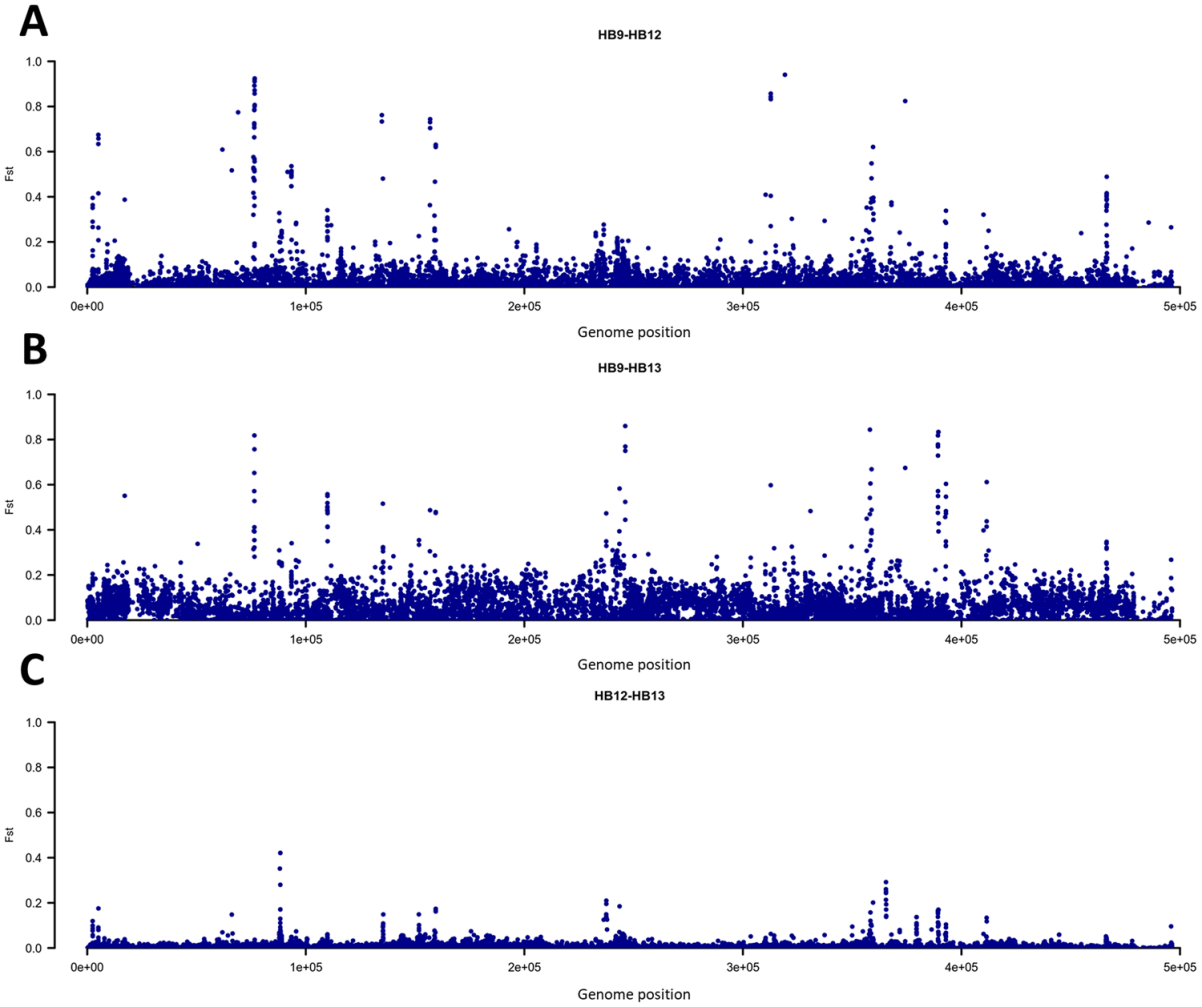
Manhattan plots of the genome-wide Fixation index (F_ST_) analyses between pair of honey samples. Each dot represents a single nucleotide polymorphism. (**A**) HB9 *vs* HB12; (**B**) HB9 *vs* HB13; (**C**) HB12 *vs* HB13.

Analysis of sequenced reads detected only one virus and one phage: (i) the *Apis mellifera* Filamentous Virus (AmFV)^65^ whose genome was highly covered and with the highest depth of sequencing among all organisms detected in this study and (ii) the *Spiroplasma phage SVTS2*, an SpV1-like plectrovirus of *Spiroplasma melliferum*, with rods with single-stranded circular DNA^68^. AmFV represented the second most abundant detected organism (about 29% of annotated reads). Its genome was almost fully covered (>99.6%) with an average sequence depth equal to 302×, 1740× and 4120× for the honey samples HB9, HB12 and HB13, respectively. The AmFV genome was characterized by the presence of 9,679 biallelic polymorphisms (~1.9% of the genome). Genetic diversity between pairs of AmFV populations (derived by the different honey samples) was investigated using the F_ST_ index (Supplementary Table S3). This analysis highlighted high similarity between the two honey samples collected from the same apiary (HB12 and HB13; F_ST_ = 0.007) and diverged largely for the HB9 sample (F_ST_ = 0.028 in the comparison with HB12; F_ST_ = 0.062 in the comparison with HB13). Figure 4 summarizes the selection signature regions identified in the three pairwise comparisons further describing the similarity/diversity reported above. In each comparison a total of ten SNPs was detected as outliers (considering the 99.9^th^ percentile). These SNPs were mainly located in intergenic regions (genic regions: AmFV_004, AmFV_0044, AmFV_106, AmFV_0111, AmFV_0158).

### Identification of equally abundant organisms across honey samples

To further characterize the honey DNA metagenomic data of the analysed samples, we identified which organisms could be considered equally abundant across samples. A total of ten organisms were detected to be equally abundant (CV% < 10.0) (Table 3), all belonged to Bacteria. Among them, the pathogen *M. plutonius* had the lowest CV% (4.7%). The top 10 list contained several honey bee gut symbionts and common microorganisms of the honey bee gut microbiota^69^. These bacteria included: (i) the Actinobacterium *Bifidobacterium asteroides*, commonly found in the gut of adult workers^37,70^, even if not at a very high level as other symbionts as also shown by the low number of assigned reads (Table 3), (ii) the gammaproteobacterium *Frischella perrara*, which causes strong activation of the host immune system^71^, (iii) the obligate fructophilic lactic acid bacterium *Lactobacillus kunkeei*, which has been shown to decrease the infection by *Paenibacillus larvae* and *Nosema ceranae*^72^, (iv) *Gilliamella apicola*, which was suggested to confer protective functions against intestinal parasites of the genus *Crithidia* and to improve dietary tolerance by the catabolic action against toxic carbohydrates^69,73^, (v) Acinetobacter spp., which is a common component of the workers gut microbiota^74^ and (vi) *Parasaccharibacter apium*, which seems to increase resistance to *Nosema* infection^75^. In addition to these microbiota components, a match with the bacteria *Erwinia gerundensis* was identified across all honey samples. This microorganism belongs to a genus that is usually associated with plant pathogens^76^. It is an epiphyte originally isolated from pome fruit trees but that has been subsequently recovered from different plant hosts in different continents, revealing its cosmopolitan nature^76^.

**Table 3:** Equally abundant organisms (Coefficient of Variation, CV% < 10) in the analysed honey samples (HB9, HB12 and HB13).

Class	Rank^a^	Organism	No. of annotated reads						
			Read counts			clr-transformed read counts			
			HB9	HB12	HB13	HB9	HB12	HB13	CV%^b^
Bacteria	species	*Melissococcus plutonius*	105,867	100,269	159,306	2.3	2.5	2.3	4.7
Bacteria	NA	*Bifidobacterium asteroides* DSM 20089	6	3	12	−2.0	−2.1	−1.9	5.4
Bacteria	species	*Frischella perrara*	161,403	128,103	169,719	2.5	2.6	2.3	5.6
Bacteria	species	*Lactobacillus kunkeei*	13,121,714	15,232,526	11,376,580	4.4	4.6	4.1	5.9
Bacteria	species	*Gilliamella apicola*	370,547	174,546	260,828	2.8	2.7	2.5	6.3
Bacteria	species	*Acinetobacter* sp. SWBY1	44	24	87	−1.1	−1.2	−1.0	7.9
Bacteria	species	*Parasaccharibacter apium*	14,090	10,745	16033	1.4	1.5	1.3	7.9
Bacteria	species	*Erwinia gerundensis*	32	12	34	−1.2	−1.5	−1.4	8.0
Bacteria	NA	*Lactobacillus kunkeei* EFB6	927,869	1,160,989	755,840	3.2	3.5	2.9	9.0
Bacteria	genus	*Lactobacillus*	565,536	674,736	447,486	3.0	3.3	2.7	9.5

^a^Level in the taxonomic hierarchy. NA indicates that the rank was not defined.

^b^Signed coefficient of variation based on the centered log-ratio (clr) transformed read counts.

Data are sorted by CV%.

### Identification of the *Apis mellifera* subspecies from honey shotgun sequencing data

Two different approaches were used to identify information that, based on shotgun sequencing data generated for each honey sample, could be useful to identify the *A. mellifera* subspecies that produced the investigated honey samples^55^. The first approach was based on the analysis of honey bee mitochondrial DNA (mtDNA) information to assign the mtDNA lineage of the honey bees^55–59^. The second approach relied on the analysis of nuclear genome informative regions containing polymorphisms that were already indicated to discriminate different honey bee subspecies and hybrid populations^60,61^.

The mining of the honey bee mtDNA-assigned sequences followed what was proposed by Utzeri *et al*.^15^, who developed a simple method based on the length of the amplified mitochondrial genome region NC_0015661:3363–3447, which discriminates different honey bee lineages (i.e. A, C and M branches). All the three investigated honey samples showed a mtDNA breadth of coverage ≥ 99.7%, with sequencing read depth in the range of 24× to 831×. The inspection of the mtDNA aligned reads from all the three samples detected only reads that could be compatible with the *A. mellifera* C lineage. None of the matching could be compatible with the A and M mtDNA lineages. These *in silico* results were also confirmed by PCR analysis of the honey extracted DNA from all three samples using the method described by Utzeri *et al*.^15^. This *in vitro* method assigned the honey samples to the C mitotype, that is the original lineage of the *A. m. ligustica* subspecies^55,56,59^.

The *A. mellifera* nuclear genome showed more variability in terms of breadth of coverage and sequencing read depth derived by the whole DNA shotgun sequencing datasets. The HB9, HB12 and HB13 honey samples had *A. mellifera* genome coverage and read depth of 98% and 10×, 22% and 0.6×, 8% and 0.4×, respectively. Only for the honey sample HB9 it was possible to obtain information for all 106 highly discriminant SNPs of the *A. mellifera* nuclear genome (a filtered subset of SNPs of the 144 ancestry-informative markers proposed by Muñoz *et al*.^60^; Supplementary Table S1). For honey samples HB12 and HB13 only 23 and 8 SNP positions could be considered, respectively (Supplementary Table S1). Therefore, these two latter samples were not further investigated for this purpose as the low number of SNPs could not provide enough information for the honey bee subspecies allocation of these samples.

**Figure 5:**
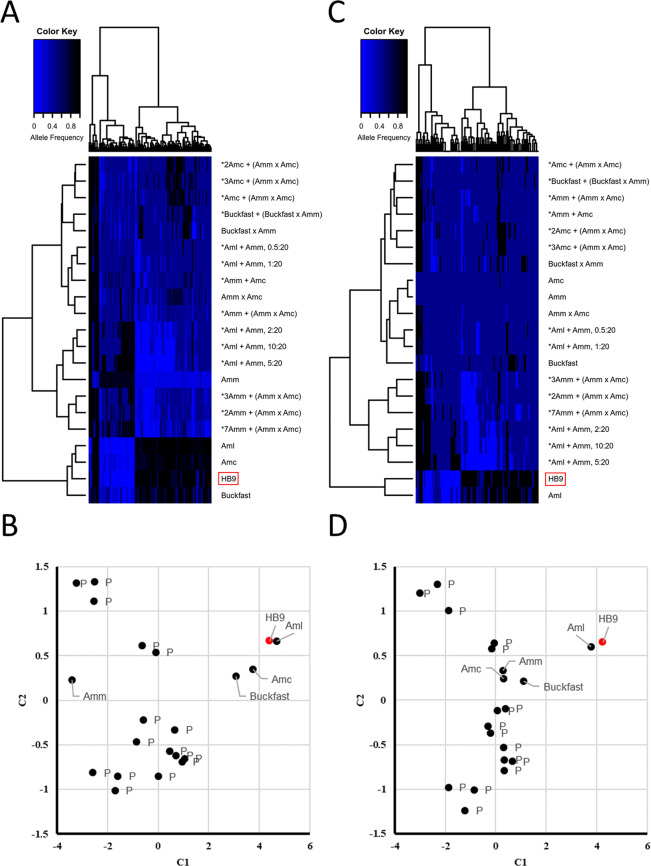
Hierarchical clustering (HCLUST) and multidimensional scaling (MDS; C1, component 1; C2, component 2) based on regular allele frequencies of the analysed honey bee populations and samples. (**A**) HCLUST and (**B**) MDS based on regular allele; (**C**) HCLUST and (**D**) MDS based on allele frequencies coded as 0/0.5/1. Names are as follows: Amm, *Apis mellifera mellifera*; Amc, *Apis mellifera carnica*; Aml, *Apis mellifera ligustica*. The star symbols indicate hybrids populations and DNA pools (P), with related dilution ratio, as reported in the original paper by Muñoz *et al.*^60^. The honey sample HB9 is highlighted in red.

Estimated allele frequencies (based on the ratio on the number of reads carrying the two alternative alleles) obtained for the 106 selected SNPs were used to cluster the honey sample HB9 with the data available from Muñoz *et al*.^60^ and Henriques *et al*.^62^ obtained from a total of 22 different subspecies or hybrid/synthetic groups of *A. mellifera* (and derived from populations of individually genotyped honey bees or from honey bee DNA pools of different subspecies). Based on allele frequency estimates, hierarchical clustering of the whole dataset evidenced two groups of honey bee populations (Fig. 5A): the first group included *A. m. carnica*, *A. m. ligustica* and Buckfast bees whereas the second group encompassed the subspecies *A. m. mellifera* and hybrids/DNA pools of honey bees with *A. m. mellifera*. Dissimilarities among populations were also demonstrated via multidimensional scaling (MDS) (Fig. 5B). Clustering analysis placed the honey sample HB9 close to *A. m. carnica* and *A. m. ligustica*. However, the inspection of the dissimilarity matrix showed closeness of the honey sample HB9 to *A. m. ligustica* (Supplementary Table S4). Clustering and MDS based on recoded allele frequencies (shrank allele frequency method; only three classes were here considered: AF = 0, AF = 0.5 and AF =1) classified the honey sample HB9 as *A. m. ligustica* (Fig. 5C,D). Reliability of these two approaches was assessed by measuring the bias of classification by means of random samples. Analyses were run on the whole dataset (22 populations) and on a subset comprising only *A. m. carnica, A. m. ligustica*, *A. m. mellifera* and Buckfast. Results are reported in Supplementary Table S5. In the first dataset, classification was biased exclusively toward the admixed/pooled populations (using both regular and shrank AFs). Removal of hybrids and DNA pools from the dataset led to classify random samples either as *A. m. carnica* (22.83%, regular AFs; 0.03%, shrank AFs) or as Buckfast (99.97%, AFs; 0.03%, shrank AFs).

## Discussion

Honey bees have proven to be useful bioindicators to detect, measure and track the source and origin of pollutants in agricultural and urban landscapes^8,77^. During their foraging and explorations activities, honey bees get exposed not only to chemicals but also to the biological components of an environment, whose traces can be transferred into the hives and then can be retrieved from the honey, the main hive product^7,9,78,79^. Honey also contains traces from the hive micro-environmental biological systems, making it an interesting matrix to disclose the complex ecological relationships among the external and internal hive environments, the hive pathosphere and, at least in part, the honey bee hologenome directly from their related footprints encoded in the honey eDNA^10,80^. Deep sequencing can describe this hologenome, represented by the collective genomes of the holobiont, which implicates organismality and mutual interactions with symbiotic relationships^11^.

A few metagenomic studies have been already focused on honey bees and their ecological biosystems with the main aim to characterize the honey bee gut microbiota and the honey bee virome^36–41^. Our previous study^10^ demonstrated that a sparse shotgun metagenomic sequencing approach focused on the honey can disclose a multi-kingdom signature that could be used for different applications, including authentication of this product, detection of the botanical origin of the honey and description of the pathosphere, among several others^10^. Deeper sequencing analyses in shotgun metagenomic investigations of honey eDNA might have the potential to estimate pathogenic indicators of honey bee colonies and this approach could become a standard evaluation tool to be applied for this purpose^81^.

As our previous sparse shotgun approach generated a few hundreds of thousands of reads, it was computationally convenient to design a strategy that relied on supervised binning based on sequence homology to annotate DNA sequences by aligning them to the NCBI nucleotide collection via BLAST^82^. This relatively simple approach could not be computationally feasible to analyse millions of reads generated by a high sequencing depth. As consequence, the current study adopted a strategy that aimed to reduce the amount of generated data to speed up sequence classification. The approach largely reduced the read set size by assembling them in longer contigs via MEGAHIT, an ultra-fast single-node assembler that overcomes the read set size problem via succinct de Bruijn graph^42^. Based on a dual 6-core processor, equipped with 42 GB of virtual memory (RAM), we assembled in less than 24 hours a total of 341,370 contigs, which represented about 95% of the sequenced reads. Contigs were then mapped to the reference databases.

However, one of the main drawbacks of the supervised binning approach is that it may rely on the incompleteness of a reference sequence database. The availability of different sources of annotation increases the probability to assign randomly sequenced metagenomic fragments, derived by the honey DNA, to regions of the genome of different organisms. Here, to maximize taxonomical classification, we used three different databases. As in our previous work^10^, one was the NCBI nt collection, populated by more than 50 million of DNA sequences encompassing all kingdoms of life. Despite this broad coverage, using this database we were able to annotate only ~7% of the assembled contigs, encompassing a total of ~5% of all sequenced reads (and ~8% of all mapped reads). These numbers are much lower than the 66% of sequenced reads that this resource was able to annotate in the previous work^10^. However, this comparison should be carefully handled as different samples were sequenced and different computational methods were applied. The sparse approach investigated monofloral honeys whereas the current study analysed polyfloral honeys. This further complicates the interpretation of the results as the reads obtained in this deep sequencing could be related to many more organisms (plants and they holobionts) that might not be represented in the reference database. Moreover, at present, we do not know the variability in sequence annotation for shotgun metagenomics in honey. Then, as different computational approaches were applied, discrepancy in number of annotated reads might be explained in part by the fact that we imposed mapping coverage of the contigs >50%, a threshold that for longer contigs cannot be achieved when relying only on the partial genome information deposited in this database for many organisms. At present the NCBI nt resource does not include the complete genome sequence for all organisms that are listed in this database. Moreover, assembly of genome sequences is prone to errors (chimerism) that in turn could affect mapping statistics.

To overcome at least in part this problem, we relied on the HoloBee database^44^, a curated database of DNA/RNA sequences from the honey bee holobiont community. This resource has been also previously used to characterize the British honey bee metagenome^37^. The HoloBee-Mop section of the database helped in the annotation of 27% of the assembled contigs, thanks to the availability of full genomes related to one protozoan, two viruses, five fungi, six metazoan and 55 bacteria^44^. In addition, through our customized database (that included the reference genome of several other organisms), we were able to annotate 66% of assembled contigs, that were mainly attributed to the *A. mellifera* and AmFV genomes. However, the genome of most eukaryotic and prokaryotic species has not been completely sequenced yet, resulting in a large fraction of reconstructed contigs that could not be mapped to a reference genome. Both these honey bee related databases were however used after the NCBI nt resource was interrogated, to avoid statistical biases that could be derived by the BLAST E-values that largely depend by the database size (the size of the HoloBee-MOP database and that of our customized database is much lower than the NCBI nt size). We preferred to have false negative calls and abundancy underestimation rather than over-estimation of holobionts.

About 26% of our assembled contigs were annotated, leading to a taxonomic profile representing 191 organisms which encompassed different groups (kingdoms or phyla): arthropods, plants, fungi, bacteria and viruses. A bias might be evident from the limited number of contigs assigned to organisms with complex and large genomes (particularly plants and fungi) that were not specifically targeted (their reference genomes were not included among the sources of annotation of our bioinformatic pipelines; for most species they are not publicly available, yet).

Data mining led us to interpret sequence information at different levels of taxonomic classification, obtaining details useful to identify similarities between samples, to characterize potential honey bee health indicators and to detect organism related markers within the multi-kingdom signatures that can be used to inform on the origin of the honey.

Comparison of the obtained taxa profiles among honey samples was done considering the compositional nature of the data: read counts for a sample is constrained by the capacity of the applied sequencing runs and therefore only relative abundance could be meaningful^51–53^. Thus, read counts were centered log-ratio transformed, capturing the relationships over the different components. Similarity between pairs of honey profiles obtained with the Aitchison’s distance^53^ and the proportionality index *ρ*^50^ indicated that the composition of honey samples HB12 and HB13 were more similar than that of these two between sample HB9. This result might derive by the same origin of the honey samples HB12 and HB13, that were both collected from the same apiary and in the same period. Therefore, these two samples have shared common environmental factors and related eDNA signatures that were then captured by the shotgun metagenomic analysis. Analysis of the *Apis mellifera* Filamentosus Virus (AmFV) genome further strengthened similarity between HB12 and HB13 samples. Spread all over the world^65,83,84^, this virus presents a large genome (~0.5 Mbp) that resulted highly variable. This feature was used to measure the closeness between pairs of honey samples by computing the F_ST_ parameter. The diversity of the AmFV genome was uncovered by taking advantage from the high sequencing depth over the whole virus genome (reported for all three samples) that could capture information about polymorphisms. F_ST_ analysis carried out on the AmFV genome confirmed the closeness between HB12 and HB13, providing a potential useful indicator to define the origin of the honey at the apiary level. AmFV is considered a weakly pathogenetic virus of honey bees which might occasionally induce colony-level symptoms when it acts with other pathogens^65,66^. However, it is not known yet if variants of this virus could have different pathogenetic effects. It would be interesting to evaluate this aspect considering also the variability we described in different regions of the reference genome of this virus (Fig. 4). Further studies are also needed to better evaluate the impact of this virus on colony health considering the relevant number of sequences that we detected that might be an indicator of high viral concentration. This virus also contributed with the largest number of reads in our previous sparse metagenomic analysis^10^ that investigated other honey samples than those analysed in this study. Adding the results from our previous study and the current one, it seems that the AmFV might constitute an important component of the honey bee holobiont community and it could be more ubiquitous than previously reported by other studies that specifically were focused on the epidemiological distribution of this virus^83–85^.

The picture derived by reads assigned to microorganisms (i.e. bacteria, fungi and protozoans) resembled a general and complex interplay and interaction between potentially honey bee pathogenic and beneficial organisms that might be present in the hive in a defined balance to prevent the manifestation of different diseases. Other microorganisms are considered disease causing agents of plant and might be useful to evaluate plant pathogenic states in the hive surrounding areas. Potential human pathogens and beneficial microorganisms were also identified.

The large number of bacterial species identified in all honey samples (bacteria accounted for 75% of all different detected species) described the complexity of the eDNA signature for this organism kingdom. Among the bacteria, the family Lactobacillaceae was the most represented in terms of number of reads and detected species. *Lactobacillus* taxa Firm-4 and Firm-5 are common and abundant honey bee gut microorganisms. Among the Firm-4 group, we identified reads from *L. mellis* and *L. mellifer* and among the Firm-5 group we identified sequences of *L. helsingborgensis*, *L. melliventris*, *L. kimbladii* and *L. kullabergensis*^36,86,87^. The largest number of bacteria reads were assigned to *L. kunkeei*, a fructophilic bee symbiont^88^, which was also the most represented bacteria in our previous study^10^. *L. kunkeei* is thought to be of environmental origin, since it is mainly found within the hive and on hive materials^37,89,90^. It has been indicated to have beneficial and protective properties on hive stability and health and might cooperate to decrease the infection by *Paenibacillus larvae* and *Nosema ceranae*^72^.

Sequence data indicated the presence of several other non-pathogenic cobiont bacteria (e.g. *Gilliamella apicola, Frischella perrara, Snodgrassella alvi*, *Bifidobacterium asteroids, Parasaccharibacter apium*), that are common components of the honey bee gut microbiota and that, on the whole, consistently contribute to more than 90% of the bacteria present in the workers’ gut^36,37,91,92^. Some of them were present in equal abundance across honey samples (i.e. *L. kunkeei*, *F. perrara* and *P. apium*) and might constitute a signature of the health condition of a colony despite the presence of other honey bee pathogenic bacteria. In addition to *M. plutonius*, *S. apis* and *P. larvae*, that were also identified in our previous metagenomic study^10^, a few other potentially pathogenic bacteria were identified in this deeper metagenomic analysis (*S. melliferum*, which together with *S. apis*, might cause a neurological disease known as “spiroplasmosis” or “May disease^67^”; *Hafnia alvei*, considered an opportunistic pathogen^93^). Surprisingly, *M. plutonius* was found to be equally abundant across the three honey samples, suggesting a widespread occurrence of this bacterium in a latent state, since colonies were healthy and did not show any symptoms of European foul brood, the disease caused by this aetiological agent. Target PCR analysis (Ribani, Utzeri, Fontanesi, in preparation) confirmed sequencing data, further supporting the ubiquitous presence of this bacterium in honey samples.

It was also interesting to note the presence of another bacterium, *Arsenophonus nasoniae* (sequence similarity around 93%). The genus *Arsenophonus* is characterized by facultative endosymbionts species with a broad host range, including several arthropods (wasps, honey bees and their parasite *V. destructor*^94,95^).

The picture related to bacteria was also enriched by the identification of a large number of reads from *Serratia symbiotica* (a total of about 0.5 millions of reads for the three honey samples, mapped on 43 contigs and SI_M_ ~ 99%; Supplementary Table S2) that is a secondary endosymbiont present in many aphids (Hemiptera: Aphididae). Some strains of *S. symbiotica* harbored by aphids of the Aphidinae subfamily are of facultative nature whereas other strains hosted by aphids of the Lachninae subfamily have established co-obligate associations with both the aphids and its primary obligate endosymbiont, *Buchnera*^96,97^. The source and origin of *S. symbiotica* in the honey is worth of further investigation. Its presence could be derived by the honeydew (that is produced by aphids) that is commonly fed by honey bees and that is used to produce honey by the workers. Honeydew signatures can be recovered in all types of honey, including blossom honey, as we recently demonstrated using a targeted metabarcoding approach that detected plant-sucking insect DNA accumulated in the honey^17^. Therefore, in this case, *S. symbiotica* could be derived by the aphid gut microbiota which might contaminate the honeydew. Another suggestive hypothesis could argue that *S. symbiotica* would be transferred directly to the honey bees through their close relationships and interdependence with aphids (via their honeydew production) derived by the feeding behaviour of the honey bees on honeydew. A high occurrence of *S. symbiotica* infection in ant populations, especially when having tended infected aphid colonies, has been recently demonstrated by Renoz *et al*.^98^. It will be interesting to evaluate if a similar transferring process of this endosymbiotic microorganism could also occur in *A. mellifera*.

The co-existence of honey bee pathogenic and beneficial fungi mirrored, to some extent, what was described for the bacteria. Among the honey bee pathogens, two fungi that infect the brood (*Ascosphaera apis* and *Aspergillus flavus*) accounted for the largest number of annotated contigs and mapped reads. However, the sampled colonies did not show any symptoms of these diseases even over the last two years (the period from which we could obtain information from the beekeepers). We also detected two unspecialized plant pathogenic fungi (*Sclerotinia sclerotiorum*, the causal agent of white mold, which has a wide host range of plants; *Rhynchosporium orthosporum*, one of the causing agent of the leaf spot disease, which mainly infects bentgrass, fescue, orchardgrass, ryegrass, and bluegrass) even if with relatively low sequence similarity (less than 90%; Supplementary Table S2). Therefore, these results could be eventually also attributed to divergent strains of these molds or to other close species. The general bias we observed against fungi species, mainly derived by the reference sequence annotation platforms (the HoloBee-MOP database included only five fungi), could be evidenced by the un-detection of several honey specialized yeasts that were not assembled in any contigs. Yeasts might be expected to be present, as we previously demonstrated by the sparse shotgun metagenomic approach^10^. Despite the NCBI nt database covers 168,908 taxa, little is known about fungal diversity that it is estimated to count 2.2–3.8 million of species^99^.

Among the honey bee health threatening agents, trypanosome infections are the least understood. Moreover, trypanosome interactions with other honey bee pathogens and consequences on host physiology and honey bee health still remain largely to be explored and disclosed. Two trypasomatids (*L. passim* and *C. mellificae*) were identified in all honey samples, with a prevalence of *L. passim* sequences and contigs than those assigned to *C. mellificae*. *L. passim* is considered the predominant trypasomatid of *A. mellifera* in Asia, Europe, North and South America and Oceania (e.g.^100^) but no epidemiological studies and distribution analyses have been carried out so far in Italy for these two protozoan parasites. It will be important to further investigate the relevance and the impact of *L. passim* and *C. mellificae* co-infection on health risks for honey bee colonies.

The description of the honey bee pathosphere was completed by the identification of other arthropods that are pests or parasites of *A. mellifera*. As expected, among this group, *Varroa destructor* contigs and sequences were the second most numerous ones after those of *A. mellifera*. Methods that so far have been developed to measure the colony infestation rate from varroa mites rely on direct count of collected mites over the counted or estimated number of workers and/or broods^101^. It would be interesting to evaluate if a ratio between *V. destructor* reads and *A. mellifera* reads obtained from honey DNA could be a reliable estimator of mite infestation of a colony. It is clear that an *in silico* determined measure is only a blind observation that should be further evaluated and interpreted considering the critical elements, biases and computational constrains. Moreover, detection and correct abundance estimation of a pathogen/parasite from a single honey sample could take into consideration potential interferences derived by different levels of pathogen loads and contamination events. However, if validated and standardized using direct measurements of mite infestation and defined with specific protocols for honey sampling, a ratio between *V. destructor* and *A. mellifera* reads could provide an interesting retrospective evaluation of mite infestation, useful to complete the pathosphere analysis from honey eDNA.

The identification of reads annotated as being derived from *Aethina tumida* from all three honey samples was quite surprising. Both adult and larvae of *A. tumida* are extremely damaging and their actions may lead to a complete structural collapse of the nest and also of the whole colony. Thus far, the small hive beetle has been reported in Italy only in two regions of the South (Calabria and Sicilia^102^). The honey samples we collected were from two apiaries of the North of Italy (Emilia Romagna region) where no reports have identified colonies infested by *A. tumida* so far. The SI_M_ of contigs assigned to this coleopter species was equal to ~87% (Supplementary Table S2). This value could leave some doubts about their correct assignment to the small hive beetle genome. However, contigs assigned to *Galleria mellonella* (a lepidopter species that is well known to be usually present in the hives without causing, in most cases, highly negative disturbance to the colony) had the same SI_M_ value of ~87%. Sequences that were assigned to the small hive beetle genome could actually belong to other beetle species that might not severely impact the honey bee colonies or that might be in close contact, in some way, with honey bees or their hives. This potential bias might be also derived by spurious matches of reads to the *A. tumida* genome because we did not include, among the compared genomes, reference sequences of all possible coleopters. A targeted analysis specifically designed to detect *A. tumida* should be able to confirm these results.

The botanical signature of the three honey samples did not indicate a high prevalence of one plant species. Therefore, they were not of monofloral origin. According to the botanical profile, these honey samples could have a polyfloral origin and might be accumulated in the honeycomb over the summer period by the practise of the nomadism, as also confirmed by the beekeepers. Nomadism is a beekeeping practise of moving apiaries to follow seasonal flowerings. Silver birch is an anemophilous species with a blooming period close or partially overlapped with many other nectarifer plants; in this situation, it is typical to have a quaternary pollen enrichment of silver birch into the hives. After the summer period, the hives were moved to hay meadows rich of alfalfa plants (*Medicago sp*.) for the winter confinement. The highest number of *Medicago* contigs (no. = 20), normally under-represented in pollens^103^, confirms the nomadic practice. Other species for which contigs have been obtained showed a low level of average similarity (SI_M_ < 93%), suggesting that in some cases only the family of the annotated species might be reliable.

The honey bee subspecies signature left into the honey was detected by recovering sequence information of the mtDNA and of the nuclear genome of *A. mellifera*. The mtDNA was fully covered by reads in all three honey samples (from 24× to 831× read depth). Inspection of the aligned reads detected the presence of only the C-mitotype, that is typical of the *A. m. ligustica* subspecies. Therefore, the *in silico* analysis of metagenomic data was able to confirm for all three honey samples what was determined *in vitro*, applying the protocol described by Utzeri *et al*.^15^. The mining of *A. mellifera* nuclear genome information was more complex and relied on the breadth of coverage and sequencing read depth on the *A. mellifera* reference nuclear genome that, in turn, resulted in the possibility to recover genotyping by sequencing data of informative SNPs. Muñoz *et al*.^60^ and Henriques *et al*.^62^ already tested the ability of a small SNP panel to allocate honey bees to a corresponding subspecies among a few that were considered, together with hybrids, Buckfast and DNA pools constituted varying concentration of DNA derived from different subspecies. In addition, these authors used this SNP panel to evaluate the level of introgression of the C-lineage within the *A. m. mellifera* subspecies. We took advantage from their results and published datasets and using two different approaches to encode SNP allele frequencies from honey DNA, coupled with two methods to measure or establish distances among datapoints, we were able to confirm the mtDNA assignment of the honey sample from which read depth was enough to obtain reliable genotyping data for most SNPs of the informative panel. The identification of the honey bee lineage and subspecies from honey has several practical applications. Conservation programmes and initiatives aiming to support locally adapted honey bee subspecies are underway in multiple European countries with the final objective to maintain *A. mellifera* biodiversity^59,104,105^. For these purposes, cost-effective molecular methods to identify purebred colonies or evaluate the level of introgression of different lineages in a population are needed to support breeding programmes and conservation actions^60,62^. The method we developed that can analyse the honey bee nuclear genome even from the honey might become an interesting alternative monitoring tool that do not need to sample the insects for these purposes. In addition, if supported by high sequencing depth, the methods we developed that identify the subspecies from the honey they produce could be useful to authenticate the entomological origin and establish marketing differentiation of hive products obtained by locally adapted honey bee genetic resources which might assure additional economic incomes to the beekeepers, as part of sustainable conservation strategies^15,105^.

The analysis of sequencing data obtained at high sequencing depth from honey DNA opened new insights into the complexity of the honey bee derived multi-kingdom signature captured by this hive product. DNA sequence information might be useful to describe the honey bee hologenome and the interplay between the honey bee superorganism and the agro-ecological environments, with several useful applications. Our methodological study demonstrated that shotgun sequencing data of honey eDNA data could be useful to define honey bee health monitoring approaches, to establish protocols to measure environmental biodiversity, to obtain information on the botanical and entomological origin of the honey that can help to define sustainable conservation programmes of the honey bee genetic resources and develop new authentication strategies of honey bee productions to defend beekeeper activities.

## Supplementary information

Supplementary information.

Supplementary information2.

Supplementary information3.

## Data Availability

The sequencing datasets generated and analysed during the current study are available in the EMBL-EBI European Nucleotide Archive (ENA) repository (http://www.ebi.ac.uk/ena), under the study accession PRJEB36075.
