# Supplementary material for: Shotgun sequencing of honey DNA can describe honey bee derived environmental signatures and the honey bee hologenome complexity
Source: Sci Rep. 2020 Jun 9;10:9279. doi: 10.1038/s41598-020-66127-1 (PMC7283317; doi:10.1038/s41598-020-66127-1)
Supplement: Supplementary file 3 — Supplementary information3. [file 41598_2020_66127_MOESM3_ESM.docx]

**Supplementary information**

**Shotgun sequencing of honey DNA can describe honey bee derived environmental signatures and the honey bee hologenome complexity**

Samuele Bovo, Valerio Joe Utzeri, Anisa Ribani, Riccardo Cabbri, Luca Fontanesi

**Supplementary Table S1**. Allele frequency of the reference alleles of the 106 highly discriminant single nucleotide polymorphisms (SNPs) in the 22 different *Apis mellifera* populations/pools reported by Muñoz et al. [^1^] and in the three analysed honey samples (HB9, HB12 and HB13).

See the Excel file **Supplementary Table S1**.

**Supplementary Table S2**. Sequencing statistics and taxonomic annotation of assembled contigs obtained in from the analysed honey samples (HB9, HB12 and HB13).

See the Excel file **Supplementary Table S2**.

**Supplementary Table S3.** Fixation index (F_ST_) distances between pairs of *Apis mellifera* Filamentous Virus populations determined by analysing single nucleotide polymorphisms defined against the reference genome of this virus.

| **Comparison** | **Mean** | **Standard deviation** | **Median** |
| --- | --- | --- | --- |
| HB9 *vs* HB12 | 0.028 | 0.067 | 0.008 |
| HB9 *vs* HB13 | 0.062 | 0.070 | 0.047 |
| HB12 *vs* HB13 | 0.007 | 0.018 | 0.002 |

**Supplementary Table S****4**. Euclidean distance, based on 106 single nucleotide polymorphisms (SNPs), between the honey sample HB9 and the different *Apis mellifera* populations/pools defined in Table S1.

| **Population/pool^1^** | **D_AF_^2^** | **D_AF-0.5_^3^** |
| --- | --- | --- |
| *2Amc + (Amm x Amc) | 4.38 | 4.53 |
| *2Amm + (Amm x Amc) | 6.42 | 5.72 |
| *3Amc + (Amm x Amc) | 4.20 | 4.66 |
| *3Amm + (Amm x Amc) | 6.58 | 6.08 |
| *7Amm + (Amm x Amc) | 7.35 | 6.63 |
| Amc | 1.93 | 4.61 |
| *Amc + (Amm x Amc) | 4.47 | 4.64 |
| Aml | 1.89 | 2.92 |
| *Aml + Amm, 0.5:20 | 4.90 | 4.77 |
| *Aml + Amm, 1:20 | 5.39 | 4.90 |
| *Aml + Amm, 2:20 | 7.16 | 6.38 |
| *Aml + Amm, 5:20 | 7.90 | 7.57 |
| *Aml + Amm, 10:20 | 7.21 | 6.82 |
| Amm | 8.15 | 4.64 |
| *Amm + Amc | 5.38 | 4.90 |
| *Amm + (Amm x Amc) | 5.69 | 5.07 |
| Buckfast | 2.69 | 4.30 |
| *Buckfast + (Buckfa | 4.36 | 4.44 |
| Amm x Amc | 5.03 | 4.77 |
| Buckfast x Amm | 4.89 | 4.90 |

^1^ Names are as follows: Amm, *A. mellifera mellifera*; Amc, *A. mellifera carnica*; *Aml, A. mellifera ligustica*. Hybrids/Pools are indicated with the star (*) symbol. For DNA pools the dilution ratio is reported (details are given in the original study by Muñoz et al. [^1^]).

^2^ Euclidean distance, based on regular allele frequencies, of sample HB9 and the different *A. mellifera* populations/pools.

^3^ Euclidean distance, based on shrank allele frequencies codes as (coded as 0/0.5/1), of sample HB9 and the different *A. mellifera* populations/pools.

**Supplementary Table S5**. Classification of 10,000 random samples to different honey bee populations or subspecies and pools.

| **Population/pool^1^** | **P_AF_^2^** | **P_AF-0.5_^3^** | **P_S-AF_^4^** | **P_S-AF-0.5_^5^** |
| --- | --- | --- | --- | --- |
| *2Amc + (Amm x Amc) | 0.39 | 0.03 | - | - |
| *2Amm + (Amm x Amc) | 0.37 | 0.11 | - | - |
| *3Amc + (Amm x Amc) | 0.65 | 0.02 | - | - |
| *3Amm + (Amm x Amc) | 0.03 | 0.02 | - | - |
| *7Amm + (Amm x Amc) | 0.00 | 0.00 | - | - |
| Amc | 0.00 | 0.00 | 22.83 | 0.03 |
| *Amc + (Amm x Amc) | 2.88 | 0.31 | - | - |
| Aml | 0.00 | 0.00 | 0.00 | 0.00 |
| *Aml + Amm, 0.5:20 | 24.70 | 34.95 | - | - |
| *Aml + Amm, 1:20 | 15.23 | 21.77 | - | - |
| *Aml + Amm, 2:20 | 0.00 | 0.00 | - | - |
| *Aml + Amm, 5:20 | 0.00 | 0.00 | - | - |
| *Aml + Amm, 10:20 | 0.00 | 0.00 | - | - |
| Amm | 0.00 | 0.00 | 0.00 | 0.00 |
| *Amm + Amc | 23.40 | 23.43 | - | - |
| *Amm + (Amm x Amc) | 15.45 | 12.73 | - | - |
| Buckfast | 0.00 | 0.00 | 77.17 | 99.97 |
| *Buckfast + (Buckfast x Amm) | 4.88 | 1.45 | - | - |
| Amm x Amc | 12.00 | 5.18 | - | - |
| Buckfast x Amm | 0.02 | 0.00 | - | - |

^1^ Names are as follows: Amm, *A. mellifera mellifera*; Amc, *A. mellifera carnica*; *Aml, A. mellifera ligustica*. Hybrids/Pools are indicated with the star (*) symbol. For DNA pools the dilution ratio is reported (details are given in the original study by Muñoz et al. [^1^]).

^2^ Percentage of random samples classified by using regular allele frequencies.

^3^ Percentage of random samples classified by using shrank allele frequencies (0/0.5/1).

^4^ Percentage of random samples classified by using regular allele frequencies. Hybrids and DNA pools were excluded from the dataset.

^5^ Percentage of random samples classified by using shrank allele frequencies (0/0.5/1). Hybrids and DNA pools were excluded from the dataset.

**Supplementary Fig. S1**. Rarefaction curves for the three analysed honey samples (HB9, HB12 and HB13).


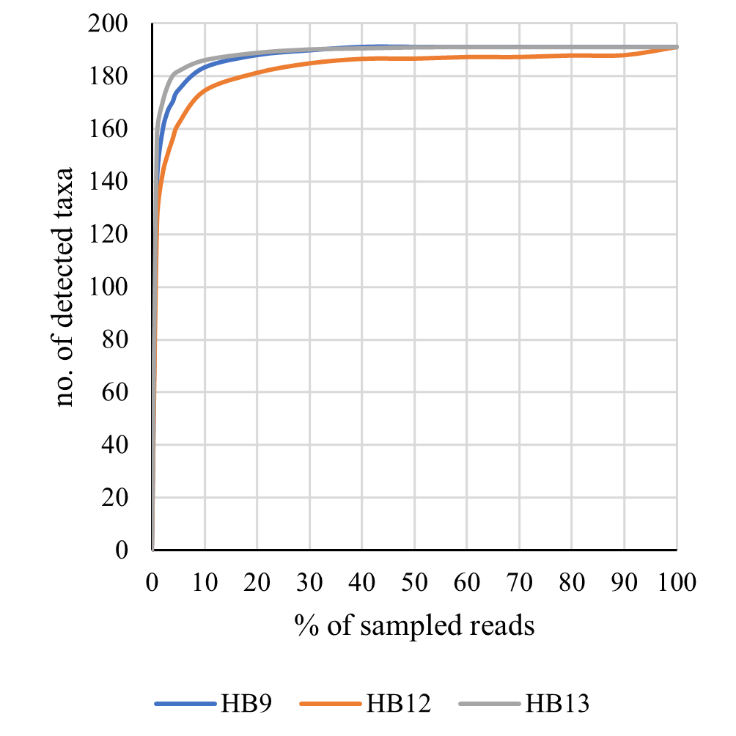


**Supplementary Fig. S2.** Scatterplots showing the relationship of read counts (centered log-ratio transformed read counts) between honey samples (HB9, HB12 and HB13). Each point identifies an organism.


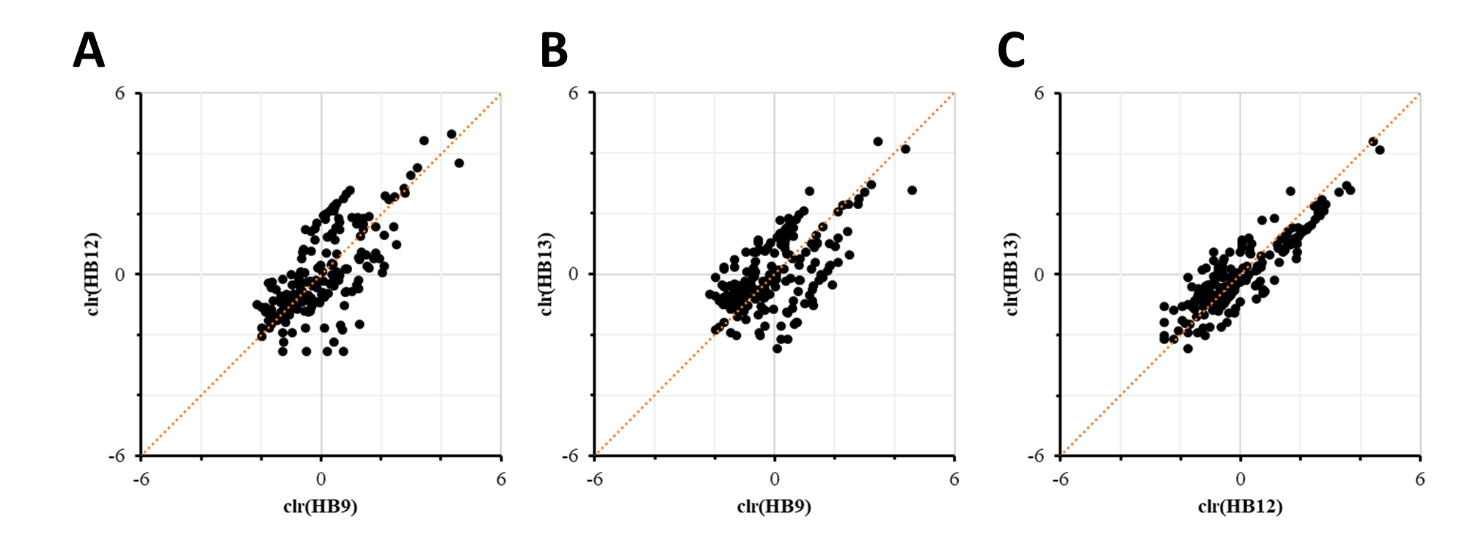


**References**

1. Muñoz, I., Henriques, D., Johnston, J. S., Chávez-Galarza, J., Kryger, P., and Pinto, M. A. 2015, Reduced SNP panels for genetic identification and introgression analysis in the dark honey bee (*Apis mellifera* *mellifera*). *PloS One*, **10**, e0124365.
